# Prevalence and Predictors of Clozapine-Associated Constipation: A Systematic Review and Meta-Analysis

**DOI:** 10.3390/ijms17060863

**Published:** 2016-06-02

**Authors:** Ayala Shirazi, Brendon Stubbs, Lucia Gomez, Susan Moore, Fiona Gaughran, Robert J. Flanagan, James H. MacCabe, John Lally

**Affiliations:** 1 GKT School of Medical Education Department, King’s College London University, London SE1 1UL, UK; ayala.shirazi@kcl.ac.uk (A.S.); lucia.gomez@kcl.ac.uk (L.G.); 2 Health Service and Population Research Department, Institute of Psychiatry, King's College London, De Crespigny Park, London SE5 8AF, UK; brendon.stubbs@kcl.ac.uk; 3 Physiotherapy Department, South London and Maudsley NHS Foundation Trust, Denmark Hill, London SE5 8AZ, UK; 4 Department of Psychosis Studies, Institute of Psychiatry, Psychology & Neuroscience, King’s College London, London SE5 8AF, UK; susan.moore@kcl.ac.uk (S.M.); fiona.p.gaughran@kcl.ac.uk (F.G.); james.maccabe@kcl.ac.uk (J.H.M.); 5 National Psychosis Unit, South London and Maudsley NHS Foundation Trust, London BR3 3BX, UK; 6 Toxicology Unit, Department of Clinical Biochemistry, King’s College Hospital NHS Foundation Trust, Denmark Hill, London SE5 9RS, UK; robert.flanagan@nhs.net

**Keywords:** constipation, clozapine, treatment-resistant schizophrenia, adverse events, systematic review, meta-analysis

## Abstract

Constipation is a frequently overlooked side effect of clozapine treatment that can prove fatal. We conducted a systematic review and meta-analysis to estimate the prevalence and risk factors for clozapine-associated constipation. Two authors performed a systematic search of major electronic databases from January 1990 to March 2016 for articles reporting the prevalence of constipation in adults treated with clozapine. A random effects meta-analysis was conducted. A total of 32 studies were meta-analyzed, establishing a pooled prevalence of clozapine-associated constipation of 31.2% (95% CI: 25.6–37.4) (*n* = 2013). People taking clozapine were significantly more likely to be constipated *versus* other antipsychotics (OR 3.02 (CI: 1.91–4.77), *p* < 0.001, *n* = 11 studies). Meta-regression identified two significant study-level factors associated with constipation prevalence: significantly higher (*p* = 0.02) rates of constipation were observed for those treated in inpatient *versus* outpatient or mixed settings and for those studies in which constipation was a primary or secondary outcome measure (36.9%) compared to studies in which constipation was not a specified outcome measure (24.8%, *p* = 0.048). Clozapine-associated constipation is common and approximately three times more likely than with other antipsychotics. Screening and preventative strategies should be established and appropriate symptomatic treatment applied when required.

## 1. Introduction

Antipsychotic medication is the mainstay of the treatment of schizophrenia, but approximately 1/3 of people with schizophrenia fail to respond to non-clozapine antipsychotics and meet the criteria for treatment-resistant schizophrenia (TRS) [1]. TRS is defined as non-response to at least two trials of antipsychotic medication of adequate dose and duration [2]. Clozapine is the only effective evidence-based treatment for TRS [3,4], and this is reflected in the clinical guidelines [2].

Despite this, clozapine remains underused, with delays of up to four years reported between the criteria for clozapine treatment being met and the first dose of clozapine [5]. One important reason for this is the side effect burden associated with clozapine. Agranulocytosis is a rare, but potentially fatal adverse effect of clozapine, with a cumulative incidence of 0.8% at 1 year and 0.38% over five years [6,7]. Regular blood monitoring is required for the prescription of clozapine in many countries. Other adverse effects include hypersalivation, metabolic disturbances including weight gain and diabetes, sedation, seizures, and constipation [8].

Constipation is difficult, incomplete, or infrequent evacuation of dry, hardened feces and may present with reduced frequency of defecation, slow stool transit time, abdominal bloating, and feelings of incomplete evacuation [9]. It is an often disregarded, but common side effect of clozapine, with a prevalence rate of 25% identified in a study of almost 500 clozapine-treated patients [10].

People with schizophrenia typically have several pre-existing risk factors for constipation, including sedentary lifestyle, obesity, reduced fiber intake, and dehydration. Clozapine reduces gastrointestinal motility, primarily via peripheral muscarinic anticholinergic activity [11], with M3 receptors in the gut wall being particularly affected [12,13,14]. This peripheral anticholinergic activity inhibits the normal innervation of gut peristalsis, resulting in a hypomotile gut. Peripheral clozapine serotonergic antagonism likely compounds the problem, as serotonin has a key role in gastrointestinal motility [15] and reduces intestinal nociception [12]. Additionally, *in vitro* studies have suggested that norclozapine, the major plasma metabolite of clozapine, may contribute to an increased risk for constipation through potent agonist activity at δ opioid receptors [16]. The antimuscarinic activity of clozapine may be more closely correlated with plasma clozapine and norclozapine concentrations than with clozapine dose [17], although it is uncertain to what degree norclozapine exerts an antimuscarinic effect [18]. Clozapine also acts as a strong histamine H 1 receptor antagonist, leading to sedation, which is another possible contributing factor to constipation.

To compound the problem, symptoms of constipation and more severe gastrointestinal hypomotility are often either not reported, or unrecognized. This may be due to patients’ unawareness of physical problems due to cognitive deficits [19], core symptoms of schizophrenia [20], reduced pain sensitivity associated with antipsychotic medication [12,21], a reduced ability to communicate discomfort [22], or the failure of some clinicians to assess and treat physical health problems [19]. Standard side effect screening tools often omit questions on constipation [23], although recently a screening specifically for clozapine, including a question on constipation [24], has been developed.

Constipation can lead to ileus, bowel obstruction, ischemic colitis, gastrointestinal necrosis, toxic megacolon, and death [25,26,27,28,29,30,31]. The incidence of clozapine-related life-threatening hypomotility from spontaneous reporting data in Australasia was 0.3% [12]. Nationwide hospital data in Denmark reports the incidence of ileus with clozapine as 0.8% [32], a similar rate to that of clozapine-induced agranulocytosis. In addition to its life-threatening consequences, constipation has a significant impact on the quality of life [33,34].

A recent narrative review [35] suggested that constipation is a common side effect of antipsychotics that is associated most frequently with clozapine treatment. However, this review was hallmarked by the paucity of studies reporting on the proportion of clozapine-treated patients with constipation (one review of five studies in China, two randomized control trials (RCTs), one observational study and two case series). Further, when comparing prevalence rates of constipation between clozapine and other antipsychotics, only one study reporting clozapine-associated constipation rates was included. Of the other identified studies, only five provided prevalence rates (one of which was for severe constipation only), and the other studies were case series or case reports. This was due to strict inclusion criteria, namely that constipation was identified as a primary outcome in antipsychotic studies. This partly reflects the reality of the lack of attention paid to this potentially fatal adverse event in clozapine trials and observational studies as a primary or secondary outcome. However, to date a systematic review has not been performed to assess either the prevalence of clozapine-associated constipation, or factors that could help predict its occurrence. Furthermore, there are no meta-analytic data comparing risk of constipation with clozapine use to other antipsychotic treatments. The use of meta-analysis is relevant as it enables the investigation of risk factors across a large number of studies and participants, distinguishing risk factors associated with clozapine-associated constipation. Further, pooling data allows for the investigation of the effect of demographic and study variables (gender, age, study setting, geographical region, study design) and clinical variables (diagnoses, method of assessment of clozapine associated constipation, duration of treatment, clozapine dosage and plasma clozapine and norclozapine concentrations, smoking, and laxative use) on the risk of clozapine-associated constipation, potentially allowing for risk stratification to be observed, which could aid clinicians in monitoring for constipation in clozapine-treated patients. Given the aforementioned gaps within the literature, we conducted a large-scale systematic review and meta-analysis.

The aim of this systematic review and meta-analysis was to assess the prevalence of constipation associated with the use of clozapine and to compare this with the prevalence associated with other antipsychotics. We anticipated completing separate subgroup analyses investigating the pooled prevalence of clozapine-associated constipation in RCTs and studies that reported on the prevalence rate of clozapine-associated constipation and conducted a comparative meta-analysis to establish if constipation is more common in people treated with clozapine *versus* other antipsychotics. We hypothesized that clozapine treatment would be associated with significantly greater risk of constipation than treatment with other antipsychotics. In addition, we sought to identify potential moderators of clozapine-associated constipation. We planned to perform subgroup analyses of the prevalence of constipation according to geographical location, study design, study setting, and the constipation assessment method used.

## 2. Methods

The review was conducted in accordance with the Meta-analysis of Observational Studies in Epidemiology (MOOSE) guidelines [36] and reported in accordance with the Preferred Reporting Items for Systematic Reviews and Meta-Analyses (PRISMA) statement [37].

### 2.1. Inclusion and Exclusion Criteria

Included in the review were: (1) studies involving adults (age 18 years or more) with a diagnosis of schizophrenia, schizoaffective disorder or a related psychotic disorder, or bipolar affective disorder according to DSM or ICD classification, treated with clozapine; (2) comparative studies, including RCTs, comparing clozapine treatment to a healthy control group or a group receiving a non-clozapine antipsychotic; (3) non-comparative (without a control group) studies; (4) comparative or non-comparative studies that reported a prevalence rate for clozapine associated constipation, not necessarily as a primary or secondary study outcome; (5) studies that defined cases of constipation by the following: patient self-report; checklist of adverse events; clinician or ICD diagnosis; consensus criteria from Rome III criteria of at least 12 weeks of two or more of the following: (i) straining during at least 25% of defecations; (ii) lumpy or hard stools in at least 25% of defecations; (iii) sensation of incomplete evacuation for at least 25% of defecations; (iv) sensation of anorectal obstruction/blockage for at least 25% of defecations; (v) manual maneuvers to facilitate at least 25% of defecations (e.g., digital evacuation, support of the pelvic floor); (vi) fewer than three defecations per week; laxative use as a proxy measure for constipation; radiological evaluation by measures such as the colonic transit test; and, (6) studies published in English in peer-reviewed journals since 1990.

Studies were excluded if (1) they did not provide sufficient data to ascertain the proportion of clozapine-treated patients with constipation; or (2) data on constipation were presented with anticholinergic data and not separately.

### 2.2. Information Sources and Searches

An electronic search using PubMed, Psychinfo, and Scopus was performed using different combinations of the following search terms: Clozapine, Clozaril, constipat*, obstipation, laxatives, Intestinal obstruction, bowel obstruction, Ileus, paralytic ileus, anticholinergic (side) effect, antimuscarinic (side) effect.

The electronic search was supplemented by a manual review of reference lists from eligible publications including two Cochrane reviews and a recent narrative review [35,38,39].

### 2.3. Study Selection

Two independent authors screened the titles and abstracts of all potentially eligible articles. Two authors applied the eligibility criteria, and a list of full text articles was developed through consensus. The two reviewers then considered the full text of these articles, and a final list of included articles was reached through consensus.

### 2.4. Data Extraction of Outcomes

The primary outcomes of interest were the point prevalence of clozapine associated-constipation, as defined in the original reports, and the odds ratio (OR) of constipation in clozapine-treated patients *versus* those treated with other antipsychotics.

Additionally, we extracted further data where possible to assess the study-level factors associated with constipation prevalence in clozapine-treated patients. The data collected from each article included: study design, geographical location, study setting, method of constipation assessment, constipation data presented as a primary or secondary outcome or as side effects of clozapine treatment, and details of clozapine participants (mean age, sex, mean dose and duration of clozapine use, mean plasma clozapine and norclozapine concentrations of the study sample, percentage of smokers, and the prevalence of those in receipt of laxatives).

To assess for differences between the prevalence of constipation based on the method of constipation assessment, a binary variable was generated where studies were either categorized as “more valid” or “less valid” in their approach to constipation assessment. Studies categorized as more valid included studies in which the diagnostic definition of constipation was assigned through clinician or ICD diagnosis, Rome III criteria, or by the proxy measure of laxative use. Studies categorized as “less valid” included studies in which constipation was self-reported or assessed through the use of checklists of adverse events.

When we identified a study that was eligible, but did not contain sufficient data to enable inclusion in the meta-analysis, we contacted the corresponding authors twice over a month to acquire the data [10,40,41,42,43,44,45,46,47,48,49,50,51].

### 2.5. Meta-Analysis

We pooled individual study data using the DerSimonian–Laird proportion method [52], which calculates pooled prevalence of constipation using inverse-variance weighted random effects meta-analysis with StatsDirect^®^ and Comprehensive Meta-Analysis^®^ software (version 3) (CMA version 2, Englewood, NJ, USA). Due to the methodological variability in the included studies, we anticipated heterogeneity, and a random effects meta-analysis was employed. We quantified any observed heterogeneity by computing the *I*^2^ statistic [53].

We assessed publication bias using the Egger [54] tests, with a *p*-value <0.05 suggesting the presence of bias. Finally, we conducted several meta-regression analyses (if *N* ≥ 4) to investigate potential moderators of clozapine-associated constipation (age, sex, clozapine dose, plasma clozapine and norclozapine concentrations, duration of clozapine use and the percentage of smokers, constipation assessment method) with Comprehensive Meta-Analysis^®^ (version 3). Despite a lack of consensus on the number of studies required for meta-regression, we chose to only conduct meta-regression where data was available from four or more studies based on recent recommendations [55].

## 3. Results

### 3.1. Search Results and Study Selection

**Figure 1:**
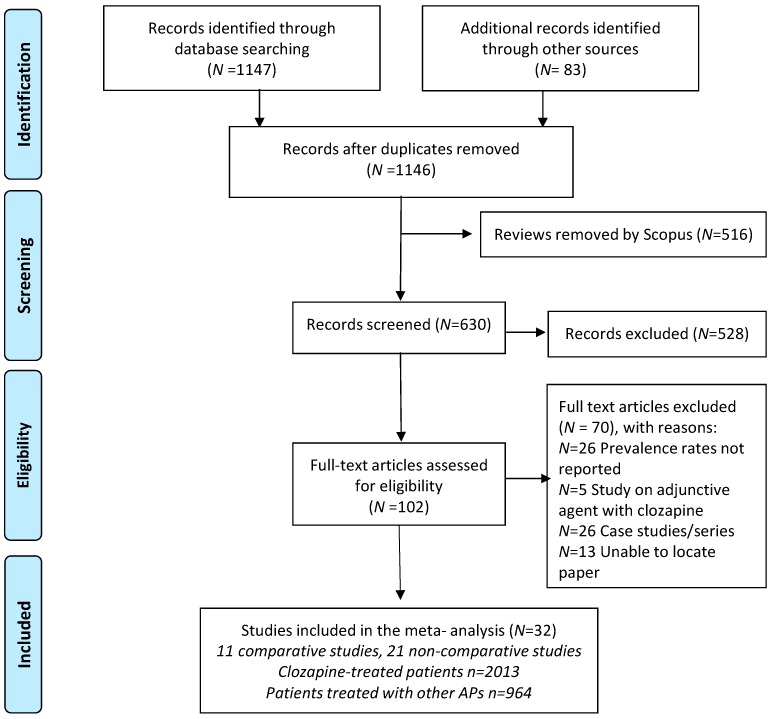
Flow diagram of article search and review process.

The initial search identified 1146 publications following removal of duplicates. Six hundred and thirty titles and abstracts were screened, indicating 102 articles for full-text viewing. After the application of the eligibility criteria, 32 of these studies were included in the systematic review. Full details of the search process are summarized in Figure 1.

### 3.2. Study and Participant Characteristics

Amongst the 32 studies included, 11 were trials that compared clozapine-treated patients to patients treated with other antipsychotics, nine of which were RCTs. Twenty-one non-comparative studies reported prevalence rates of clozapine-associated constipation. In total the dataset included 2013 patients treated with clozapine (comparative studies *n* = 935, non-comparative studies *n* = 1078) and 964 patients treated with other antipsychotics. Details on the comparative and non-comparative studies included and the participants in these studies reporting prevalence rates for clozapine-associated constipation are presented in Tables S1 and S2, respectively.

#### 3.2.1. Included Studies—Non Comparative Studies

The sample size of the studies ranged from 15 [56] to 202 [57]. The mean age (data unavailable from [58,59]) of participants was 39.2 years (range: 28.2 [60]–47.6 years [61]) and 71% were male (gender data unavailable from [59]). The mean clozapine dose used (data missing from [59]) was 381 mg (SD = 138 mg). The mean plasma clozapine concentration was 0.46 (SD = 0.26) mg/L and the mean plasma norclozapine concentration was 0.29 (SD = 0.17) mg/L.

This sample of non-comparative studies reporting prevalence rates of clozapine-associated constipation included three RCTs [62,63,64]. This included one crossover study that compared two different clozapine preparations (oral FazaClo *versus* Clozaril tablets) [62] and two studies that compared the use of clozapine alone to the use of clozapine augmented with another medication (*n* = 1 augmented with risperidone, *n* = 1 augmented with minocycline). For our study, we extracted data from the Clozaril-alone arm of the crossover study [62], as the prevalence of constipation within both arms over the study was unclear and this arm provided more data. Additionally, eight prospective studies [17,49,58,61,65,66,67,68], six retrospective cohort studies [48,50,56,57,59,69], and four cross-sectional studies [24,70,71,72] were included. Of the 21 studies that reported on the prevalence rate of clozapine-associated constipation, one was carried out across more than one geographical region [63]. Five studies were carried out in Europe [24,57,63,68,70], seven in North America [17,45,50,59,60,63,64,65,72], six in Asia [49,56,58,61,63,66], two in Africa [62,71], one in the Middle East [48], and one in Oceania [69]. Nine studies were conducted in in-patient settings (*n* = 395) [17,48,49,50,58,59,60,61,65], 10 involved outpatients (*n* = 627) [24,56,57,62,66,68,69,70,71,72], and two included patients from mixed settings (*n* = 56) [63,64].

Five of the studies assessed for constipation or adverse effects of clozapine as a primary outcome [24,57,59,70,71], while six other studies assessed for these as secondary outcome measures [17,49,56,60,68,71]. Six studies used self-reports from patients to measure for constipation [48,50,59,62,64,65], nine studies used a checklist to determine the presence or absence of constipation in participants [17,24,56,60,63,66,69,70,72], and four screened for constipation using structured clinical assessments [49,61,68,71]. One study identified constipation using laxative prescription rates in patients [57], with only one other study reporting on laxative use [71].

#### 3.2.2. Included Studies—Comparative Studies

The sample size of these studies ranged from 20 [73] to 956 [10]. The mean age of clozapine-treated participants (37.5 years (age range 28.6 [74] to 46.1 years [51]) was similar to that of patients treated with other antipsychotics (37.5 years (age range: 28.4 [74] to 41.7 years [51]). Sixty-nine percent of those treated with clozapine and 65% of those treated with other antipsychotics were male. The mean clozapine dose in clozapine-treated patients (from 10 studies (data missing from [74])) was 336 mg (SD = 144 mg). Only one study reported plasma clozapine concentrations (0.49 (SD = 0.14) mg/L) [75] and none of the comparative studies reported plasma norclozapine concentrations.

Of the 11 comparative studies, three were carried out across several geographical regions [10,76,77]. Five studies were conducted in North America [10,76,78,79,80], five in Europe [10,73,76,77,81], two in South America [10,51], two in Africa [10,77], one in Oceania [75], and one in Asia [74]. Five studies were conducted in in-patient settings (*n* = 263) [73,74,75,80,81], four involved out-patients (*n* = 1186) [10,51,78,79], while two studies included participants from mixed settings (*n* = 450) [76,77]. Constipation was the primary outcome in two studies [51,75], and clozapine-related side effects were the focus of one study [80]. Three studies measured clozapine-related side effects as a secondary outcome [74,76,77]. Five studies determined constipation rates through self-reporting by participants [10,73,76,77,81], while three studies used checklists [74,79,80], one used a structured clinical assessment [78], and two [51,75] used the Rome III criteria to measure constipation rates.

From the Baptista study [51] we included the constipation rates determined using Rome III diagnostic criteria of 33.3% in clozapine-treated patients and 12.5% in patients treated with other antipsychotics (personal communication). This study also objectively assessed for gut hypomotility using a colonic transit diagnostic test. Gut hypomotility was identified in 51% of clozapine-treated patients compared to 31% in those treated with other antipsychotics, with no significant clozapine dose relationship identified. In the Every–Palmer study [75], constipation was assessed for in several ways. The data included in our meta-analysis were constipation rates identified using the Rome III diagnostic criteria, with constipation identified in 57.9% of clozapine-treated patients compared to 23.5% of patients treated with other antipsychotics. However self-reporting in this study sample identified constipation in only 20% of clozapine-treated patients compared to 17.6% of patients treated with other antipsychotics, while laxative use was observed in 84.2% of clozapine-treated patients compared to 17.6% of patients treated with other antipsychotics. Finally, the use of a colonic transit diagnostic test identified colonic hypomotility in 80% of clozapine-treated patients, compared to 0% of patients treated using other antipsychotics.

## 4. Meta-Analysis

### 4.1. Prevalence of Constipation on Clozapine

Full details of the meta-analysis results are summarized in Table 1. It was possible to pool data from 32 studies to establish that the point prevalence of clozapine-associated-constipation (*n* = 2013) was 31.2% (95% CI: 25.6–37.4, *I*^2^ = 84%, 32 studies), with no evidence of publication bias (Egger = −0.82, *p* = 0.33).

Significantly higher rates of constipation were observed in inpatient settings (40.5%, 95% CI: 31.4–50.4, studies = 14, *p* = 0.02) than in outpatient (26.2%, 95% CI: 19.2–34.3, studies = 14) and mixed settings (22.2%, 95% CI: 12.2–36.8, studies = 4). The mean clozapine dose used in the studies from inpatient settings (*n* = 12) was 396 (SD = 134) mg/day, which was not significantly higher than the mean dose used in outpatient settings (*n* = 14) (mean dose = 338 (SD = 113) mg/day (*t* = 1.201, *p* = 0.241). There were no significant differences between mean serum clozapine (*t* = 1.455, *p* = 0.205) and mean norclozapine concentrations (*t* = 2.487, *p* = 0.089) observed between inpatient and outpatient settings. No significant difference in constipation was observed according to geographical region or study design (full details in Table 1).

Additionally, there were no significant differences observed in the prevalence of clozapine-associated constipation between studies that used less valid methods to assess for constipation (30.6%, 95% CI: 23.9–38.2, studies = 23, *I*^2^ = 85%, *p* = 0.79) compared to studies using more valid assessment methods (32.5%, 95% CI: 22.0–45.1, *I*^2^ = 79%).

Contrastingly, significantly higher rates of constipation were observed in studies that described constipation rates or side effects as a primary or secondary outcome measure (36.9%, 95% CI: 28.7–44.8, studies = 18, *I*^2^ = 86%, *p* = 0.048) compared to studies that did not include them as specified outcome variables (24.8%, 95% CI: 17.8–33.4, *I*^2^ = 65).

### 4.2. Prevalence of Constipation in Clozapine versus Other Antipsychotics

**Figure 2:**
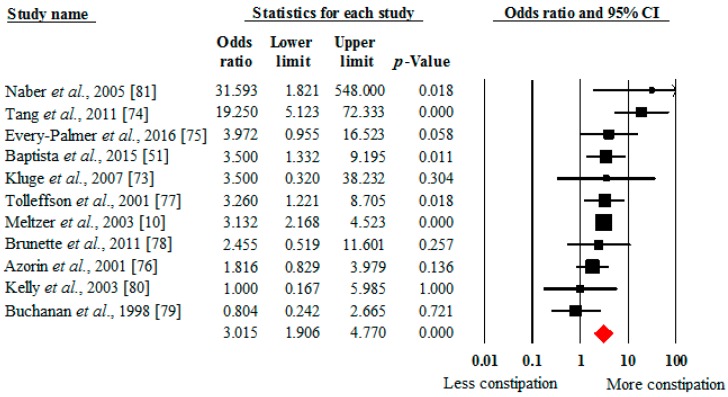
Odds of constipation in people taking clozapine *versus* other antipsychotics. Black dot = study summary results of mean and standard deviation. Red dot = Pooled summary mean and standard deviation effect size.

A comparative meta-analysis established that clozapine-treated patients (*n* = 935) were three times more likely to have constipation (OR 3.02, 95% CI: 1.91–4.77, *p* < 0.001, 11 studies, *I*^2^ = 45%) than those treated with other antipsychotics (*n* = 964) (Figure 2). The Egger bias test (0.24, *p* = 0.75) did not indicate any publication bias.

**Figure 3:**
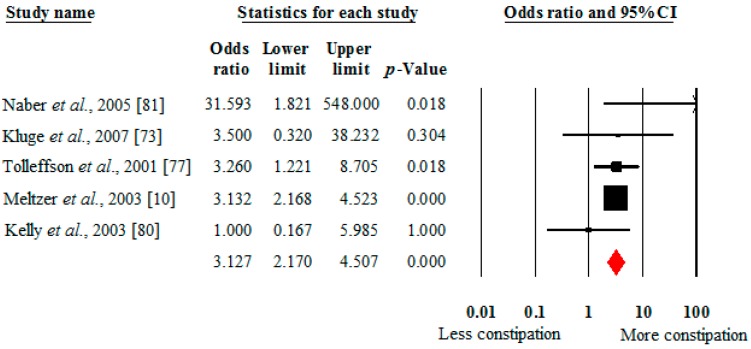
Odds of constipation in people taking clozapine *versus* olanzapine.

As shown in Figure 3, the risk for clozapine-associated constipation was greater for individuals prescribed clozapine compared to those prescribed olanzapine (OR 3.13, 95% CI: 2.17–4.51, *p* < 0.001, 5 studies, *I*^2^ = 2%). The Egger bias test (0.24, *p* = 0.79) did not indicate any publication bias. There were insufficient data to compare the risk between clozapine and other individual antipsychotics. The individual studies demonstrated an increased risk for constipation in one study for clozapine (14%) compared to risperidone (8.2%) [76], and in another study comparing clozapine (73.3%) to quetiapine (12.5%) [74]. A slightly increased rate of constipation was identified for haloperidol-treated patients (19%) compared to clozapine-treated patients (16.1%) in another study [79].

### 4.3. Moderators of Clozapine-Associated Constipation across All Studies

Full details of the meta-regression analyses are presented in Table 2. Briefly, meta-regression analyses did not identify any significant predictors of clozapine-related constipation.

Clozapine-associated constipation was not significantly related to the percentage of male patients (β = 0.0081, 95% CI = −0.0071–0.0233, *p* = 0.30, *R*^2^ = 0), mean age (β = 0.0090, 95% CI = −0.0513–0.0693, *p* = 0.77, *R*^2^ = 0.08), mean clozapine dose (β = 0.0017, 95% CI = −0.0004–0.0039, *p* = 0.11, *R*^2^ = 0), mean plasma clozapine concentration (β = 3.0455, 95% CI = −0.6171–6.708, *p* = 0.10, *R*^2^ = 0.02), mean plasma norclozapine concentration (β = 3.3561, 95% CI = −1.6898–8.4021, *p* = 0.19, *R*^2^ = 0.01), duration of clozapine treatment (β = 0.0013, 95% CI = −0.0024–0.005, *p* = 0.49, *R*^2^ = 0), and percentage of smokers (β = 0.0241, 95% CI = −0.0039–0.0522, *p* = 0.0918, *R*^2^ = 0, *n* = 8 studies).

## 5. Discussion

### 5.1. General Findings

This is the first systematic review and meta-analysis to investigate clozapine-associated constipation, and provides the most comprehensive assessment of the prevalence of and risk factors for this condition. Our study identified that 31.2% of clozapine-treated patients had constipation when surveyed. Our findings also established that constipation is three times more likely in those treated with clozapine compared to treatment with other antipsychotics (OR 3.02, *p* < 0.001). Further, we identified a threefold increased likelihood of constipation in those treated with clozapine compared to olanzapine (OR 3.13, *p* < 0.001).

From the subgroups analyses, significantly higher rates of constipation were observed in those treated in inpatient compared to outpatient or mixed settings. The variation in constipation rates by study setting could not be explained by variations in mean clozapine doses or plasma clozapine and norclozapine concentrations. A possible contributory factor may have been increased vigilance among clinical staff in inpatient settings, with the use of monitoring tools such as the Bristol Stool Chart and questioning about constipation as part of routine clinical practice, which may have led to an increased subjective awareness of constipation in patients. Variation in lifestyle factors such as diet and exercise habits may have further contributed to this difference in the reported incidence of constipation, but it was not possible to study this further from the data available. We found no significant differences in constipation rates according to study design or geographical region.

In meta-regression analyses, age, sex, diagnosis, percentage of smokers, duration of treatment, clozapine dose, and plasma clozapine and norclozapine concentrations did not predict constipation. Some of these results, in particular clozapine dose and plasma clozapine/norclozapine concentrations, are surprising.

Our systematic review identified 11 eligible controlled studies published between 1998 and 2016. However, we also identified that scant attention is paid to this adverse event in clozapine RCTs as constipation rates are often either not reported, or not reported separately from anticholinergic group effects. This mirrors the previously identified underreporting of anticholinergic side effects in antipsychotic randomized trials [82]. Given the high prevalence of clozapine-associated constipation identified in this meta-analysis, the failure of clozapine RCTs to systematically assess for this adverse event needs to be addressed, as does the variable rates of screening for constipation in clinical practice. Notably, very few studies have assessed clozapine-associated constipation as a primary outcome, or, with the exception of case reports, investigated its prognostic implications. Future research would benefit greatly if standardization for the reporting of constipation could be reached, both in its inclusion as an adverse event outcome and its method of measurement.

### 5.2. What this Study Adds

A previous narrative review of antipsychotic use and constipation concluded that constipation is most frequently associated with clozapine [35]. Our meta-analysis builds on this conclusion in a number of ways.

Firstly, we were able to pool data from 11 studies and establish that the prevalence of constipation is significantly increased in clozapine-treated patients compared to those treated with other antipsychotics; Secondly, we have established that the pooled prevalence is 31.2%, higher than the 14.7%–17.1% identified in studies of the general population [83,84], particularly since these studies showed higher prevalence rates in older people, compared to the younger mean age of people identified in our study; Thirdly, we were able to conduct meta-regression analyses to investigate potential moderators for these relationships. However, due to the paucity and inconsistency of the reporting of possible risk factors, in particular plasma clozapine concentrations, our moderator analyses did not enable us to identify any study-level predictors for clozapine associated constipation, other than study setting. However, it is important to emphasize that the lack of associations between studies does not constitute evidence of a lack of association at the patient level within each study. For example, while we did not find an association between constipation risk and mean clozapine dose or plasma clozapine concentration at the study level, this does not preclude the existence of associations at the patient level within each study.

### 5.3. Clinical Implications

This study has highlighted the high rates of constipation that occur with clozapine compared to other antipsychotic medication. Constipation, if untreated, can lead to life-threatening complications [13]. The use of clozapine in schizophrenia is associated with a doubled risk for ileus (OR 1.99, 95% CI: 1.21–3.29) and a 7-fold increased risk for fatal ileus (OR 6.73, 95% CI: 1.55–29.17) compared with other psychotropic medication [32,85], adverse events that are preceded by constipation.

A systematic review (Cohen *et al.*, 2012) indicated that while the incidence of agranulocytosis is 0.95–2 times more likely than gastrointestinal hypomotility, the case-fatality rate of gastrointestinal hypomotility of 15.0%–35.7% is estimated to be approximately 10 times higher than that of agranulocytosis (case fatality rate of 2.2%–4.2%). A reason for the reduced case fatality with clozapine-induced agranulocytosis compared to that associated with clozapine-induced gastrointestinal hypomotility is likely related to the early recognition of the condition from the regular hematological monitoring required in many countries, a systemic screening approach that has yet to be incorporated for the monitoring of constipation.

Despite this increased case fatality rate secondary to gastrointestinal hypomotility, and the high rate of constipation identified in our meta-analysis, clozapine treatment guidelines rarely mention constipation, and few recommend screening patients for constipation [35] or give advice on the active management of it when it occurs. To our knowledge, the only current guidelines to recommend screening are Dutch guidelines for the monitoring of physical health with antipsychotic use [86] and the Maudsley Prescribing Guidelines [87], although recently a screening specifically for clozapine-related adverse effects, including a question on constipation, has been developed [24].

Case reports have highlighted the sudden onset of life-threatening symptoms due to undetected constipation in clozapine-treated patients, with few reporting constipation to their clinicians before-hand [25]. This could be due to either insufficient monitoring or to an impaired ability to communicate symptoms [22], reduced pain sensitivity possibly being a factor here [21]. Each component should be considered when assessing constipation in clozapine-treated patients.

Not only is the outcome from untreated constipation potentially fatal, but as a medication side effect it can contribute to treatment non-adherence, which can have a particularly devastating impact on clinical stability in clozapine-treated patients [88]. Constipation in the general population is considered an important health problem, with well-established diagnostic criteria [89], and it has been shown to affect both socioeconomic and clinical outcome as well as subjective health-related quality of life [34]. However, there has not been a similar level of acknowledgment for clozapine-related constipation.

Our findings reinforce the need for active screening for constipation in clozapine-treated patients. Firstly patients, staff and community carers or family members must be educated on the risk of constipation and informed of the possible complications of untreated constipation. When prescribing clozapine, the concurrent use of medications known to increase the risk of constipation should be minimized. Specific attention should be paid to the use of anticholinergic treatments used for clozapine-related hypersalivation. Further, caution should be taken in those with a history of gastrointestinal disease or lower abdominal surgery. Changes in smoking habit should be noted, and the clozapine dose adjusted accordingly [90]. Clozapine is largely metabolized by cytochrome P (CYP) 1A2 enzymes. Polycyclic aromatic hydrocarbons in cigarette smoke are a potent inducer of CYP1A2, and this high inducibility of CYP1A2 in smokers leads to increased clozapine metabolism and decreased plasma concentrations [90,91]. The effect of starting or stopping smoking is important, in that non-smokers who start smoking risk losing the benefit of clozapine in two to three days; conversely, those who stop smoking are at risk of clozapine toxicity, which may include seizures, unless the dose is adjusted promptly. Further, those who stop smoking are already at an increased risk of constipation due to a reduction in bowel motility [92], and this allied to the increased plasma clozapine concentrations that occur without dose reduction may put individuals at increased risk of clozapine-associated constipation.

Protocols for monitoring constipation in clozapine-treated patients should be implemented when clozapine initiation takes place. This may utilize the Bristol stool chart, a widely used system to monitor stool frequency and consistency [93]. Clinicians as part of routine clinical practice should actively ask about symptoms of constipation, including the frequency and difficulty of defecation.

Two of the studies included in this review objectively measured gastrointestinal hypomotility using colonic transit tests. The diminished intestinal propulsive activity seen with hypomotility appears to be an underlying mechanism for clozapine-associated constipation and its associated morbidity and mortality. Both these studies found high rates of gastrointestinal hypomotility (50%–80%). Of the 51% individuals objectively hypomotile in one of these studies, 43.5% were negative for constipation diagnosed using the Rome III criteria [51]. In the other study [75], while 80% of the sample showed gastrointestinal hypomotility, only 58% of the same sample were positive for constipation using the Rome III criteria, and only 20% of individuals self-reported constipation. Findings from both studies indicate that constipation symptoms may not be sufficiently sensitive predictors of objective pathology.

Clozapine-treated patients should be regularly advised to increase their fluid and fiber intake, and physical activity as an aid to prevent constipation. More liberal use of laxatives, even in combination, for constipation may be necessary to prevent the progression of constipation. In the case of mild constipation, a bulking agent may be sufficient. However, where symptoms persist, a stool softener or laxative may be used [12]. Where constipation is more severe, stimulant cathartics, such as senna, or enemas may be required. Some other treatments found to be effective include lubiprostone [85], orlistat [94], and bethanechol [95]. However, despite the common use of laxatives and other treatments for constipation in the general population, robust, well-replicated evidence for their effectiveness is lacking [96]. These deficits in the literature are more pronounced in interventions for clozapine and other antipsychotic-related constipation, where no systematic review of treatment interventions has been performed to date [97]. However, we advocate that the liberal use of laxatives is appropriate, either singly or in combination.

If patients present with severe abdominal pain, they should be referred for a medical or surgical assessment to exclude intestinal pathology, which can rapidly lead to death. Surgical intervention may be required. Ultimately, clinicians must be made more aware of the hazards associated with constipation to minimize its potential consequences; early screening and monitoring should decrease constipation rates to improve the comfort, satisfaction, and adherence of patients, and avoid the progression to a stage requiring surgery.

### 5.4. Future Directions

There is a need for evidence-based research on the management of constipation in clozapine treated patients. Future research must address possible preventative measures, both lifestyle and pharmacotherapeutic interventions. Additionally, our study has indicated the need for clinical guidelines to be updated to reflect the need for systematic monitoring and treatment of clozapine-associated constipation.

### 5.5. Limitations

Firstly, there were a wide variety of assessment methods used to record constipation, which included patient self-reported, side effects checklists, clinician diagnosis, Rome III criteria, or using laxative prescription rates to ascertain rates of constipation. Only two studies [51,75] measured constipation using the Rome III criteria. The lack of use of methods of assessment such as the Rome III criteria, which is a preferred method to assess for constipation, is a limitation of our review and meta-analysis, although we did not identify a significant difference in the prevalence rates of constipation identified in studies using more and less valid assessment methods to diagnose constipation.

Secondly, there were a limited number of studies reporting important moderators such as plasma clozapine and norclozapine concentration. In particular, this limitation may suggest that our finding of a lack of association between increased plasma clozapine concentrations and constipation may have been a type II error. Further, it is important to consider a limitation of meta-regression analyses across studies, in that that the relationship between constipation and the average patient plasma clozapine concentrations from across studies may not be the same as the relationship with individual patient plasma concentrations from within studies, particularly when averages of patient characteristics in each study were used as covariates in the regression [98].

Thirdly, only six studies focused on constipation as a primary outcome or secondary outcome. Four studies focused on clozapine side effects (including constipation) as a primary outcome, while eight studies included side effects (including constipation) as a secondary outcome. Fourteen studies did not measure either constipation or side effects as specified outcome measures. Meta-analysis showed significantly higher rates of constipation in studies that listed either constipation or side effects as an outcome variable (36.9%, *p* = 0.048) compared to studies that did not (24.8%). The higher rates identified in those studies may reflect a truer estimate of the prevalence of clozapine-associated constipation, and the prevalence of constipation identified in this meta-analysis may be an underestimate. However, our work in providing a more comprehensive review of the literature has identified higher prevalence rates than the rate of 21.3% identified by De Hert *et al.* [35], and likely offers the most valid estimation of the rate of clozapine-associated constipation in the literature.

Further, there was limited information on other potentially important moderators of constipation such as physical activity and diet. Additionally, high heterogeneity was found across the studies included in the meta-analysis and disparate sample sizes were identified, though heterogeneity was evenly distributed across studies with different assessment methods.

## 6. Conclusions

Our systematic review and meta-analysis have demonstrated an increased risk of constipation in people treated with clozapine. Clozapine-associated constipation is a more frequent adverse effect than is recognized by many clinicians. Awareness of this important side effect needs to increase, because early detection and management are key to minimizing risk. We did not identify a relationship between plasma clozapine concentrations and constipation, though this was likely due to a paucity of studies reporting this variable. There is a need for future prospective research to better understand this relationship and the implications of smoking cessation without a corresponding decrease in clozapine dose. Future research focusing on interventions for clozapine-associated constipation is needed. Screening and preventative strategies should be established and appropriate symptomatic treatment applied when required.

## Figures and Tables

**Table 1:** Subgroup analyses of moderators of constipation in people treated with clozapine.

Analysis	Number of Study Estimates	Meta-Analysis				Heterogeneity	Publication Bias
		Prevalence	95% CI		Between Group *p* Value	*I*^2^	Egger Test (*p* Value)
**Main analysis**	32	31.2	25.6	37.4		84	−0.82 (*p* = 0.33)
**Geographical region**					0.18		
*Europe*	6	27.7	17.4	41.0		80	0.65 (*p* = 0.46)
*North America*	10	41.6	30.6	53.4		72	−0.78 (*p* = 0.26)
*South America*	1	33.3	10.8	67.4		0	N/A
Asia	6	35.0	21.8	50.9		88	−0.66 (*p* = 0.37)
*Oceania*	2	36.9	16.0	64.2		80	N/A
*Africa*	2	24.6	9.6	50.1		89	N/A
*Middle East*	1	11.9	2.9	37.7		0	N/A
*Various*	4	19.5	10.8	32.7		62	−0.56 (*p* = 0.44)
**Setting**					0.02		
*Inpatient*	14	40.5	31.4	50.4		87	−0.66 (*p* = 0.46)
*Outpatient*	14	26.2	19.2	34.3		75	−0.771 (*p* = 0.29)
*Mixed*	4	22.2	12.2	36.8		70	0.33 (*p* = 0.40)
**Study design**					0.31		
*Cross sectional*	6	40.7	27.4	55.3		42	0.49 (*p* = 0.75)
*Retrospective*	6	28.2	17.2	42.6		81	−0.51 (*p* = 0.67)
*Prospective*	8	34.4	23.3	47.6		85	−0.44 (*p* = 0.23)
*Randomized control trial*	12	25.9	18.3	35.4		80	−0.88 (*p* = 0.46)
**Constipation method**					0.70		
*Self-reported*	11	26.7	18.2	37.2		59	1.13 (*p* = 0.15)
*Checklist*	12	34.7	24.7	46.4		90	−0.59 (*p* = 0.33)
*Clinician diagnosis*	6	28.4	16.5	44.1		81	0.419 (*p* = 0.09)
*ROME III*	2	43.1	11.1	70.1		62	N/A
*Laxative use*	1	35.2	11.1	70.3		0	N/A

N/A: Publication bias was only conducted where data was available from four or more studies.

**Table 2:** Meta-regression of moderators of constipation.

Moderator	Number of Studies	β	95% CI		*p* Value	*R*^2^
*Mean age*	30	0.0090	−0.0513	0.0693	0.7704	0.08
*Percentage of males*	31	0.0081	−0.0071	0.0233	0.2953	0
*Percentage of smokers*	8	0.0241	−0.0039	0.0522	0.0918	0
*Clozapine mean dose*	30	0.0017	−0.0004	0.0039	0.1085	0
*Plasma clozapine*	9	3.0455	−0.6171	6.708	0.1032	0.02
*Plasma norclozapine*	7	3.3561	−1.6898	8.4021	0.1924	0.01
*Number of weeks clozapine treatment*	28	0.0013	−0.0024	0.005	0.4888	0
*% Sample schizophrenia*	27	−0.0020	−0.0161	0.0121	0.7825	0
*% Sample schizoaffective disorder*	11	−0.0009	−0.0223	0.0204	0.9332	0

## Supplementary Materials

Supplementary materials can be found at http://www.mdpi.com/1422-0067/17/6/863/s1.

## Abbreviations

TRS	Treatment-resistant schizophrenia
MOOSE	Meta-analysis of Observational Studies in Epidemiology
PRISMA	Preferred Reporting Items for Systematic Reviews and Meta-Analyses
DSM	Diagnostic and Statistical Manual of Mental Disorders
ICD	International classification of diseases
RCT	Randomized controlled trial
OR	odds ratio
CYP	Cytochrome P
