# Supplementary material for: Prevalence and Predictors of Clozapine-Associated Constipation: A Systematic Review and Meta-Analysis
Source: Int J Mol Sci. 2016 Jun 2;17(6):863. doi: 10.3390/ijms17060863 (PMC4926397; doi:10.3390/ijms17060863)
Supplement: Supplementary file 1 [file ijms-17-00863-s001.pdf]

Supplementary Materials: Prevalence and Predictors of Clozapine-Associated Constipation: A Systematic Review and Meta-Analysis

Ayala Shirazi, Brendon Stubbs, Lucia Gomez, Susan Moore, Fiona Gaughran, Robert J. Flanagan, James H. MacCabe and John Lally

Table S1. Summary of included studies comparing the prevalence of constipation among clozapine-treated patients against patients treated using other antipsychotics.

Study	Location, Design, Setting, and Constipation Assessment Method	Clozapine-Treated Patients, *N*, Age, % Male	Other Antipsychotics-Treated Patients, *N*, Age, % Male	Mean Clozapine Dose (mg) ± SD *	Number (%) Clozapine-Treated Patients with Constipation	Number (%) Patients with Constipation in Other Antipsychotics Group
Azorin *et al.*, 2001 [76]	Canada and France, RCT, Mixed in- and out-patients, Self-reported	*N* = 136, 37.8 ± 10.4 years, 77% male	*N* = 134, 39.5 ± 11.3 years, 65.4% male	642 ± 212	19 (14%)	11 (8.2%)
Baptista *et al.*, 2015 [51]	Venezuela, Cross-sectional study, Out-patients, Rome III criteria	*N* = 45, 46.1 ± 11.9 years, 66.7% male	*N* = 64 41.7 ± 13.3 years, 40.6% male	155 ± 86	15 (33.3%)	8 (12.5%)
Brunette *et al.*, 2011 [78]	USA, RCT, Out-patients, Clinician diagnosis	*N* = 15, 33.3 ± 10.0 years, 80% male	*N* = 31, 39 ± 10.2 years, 75% male	319 ± 163.5	4 (26.7%)	4 (12.9%)
Buchanan *et al.*, 1998 [79]	USA, RCT, Out-patients, Adverse events checklist	*N* = 38, 41 ± 6.4 years, 65.8% male	*N* = 37, 40.1 ± 7.9 years, 73% male	415 ± 50.7	6 (16.1%)	7 (19%)
Every-Palmer *et al.*, 2016 [75]	New Zealand, Cross-sectional study, In-patient study, Rome III criteria	*N* = 20, 37 ± 8.2 years, 70% male	*N* = 17, 42 ± 10.9 years, 88.2% male	528 ± 161	11 (57.9%)	4 (23.5%)
Kelly *et al.*, 2003 [80]	USA, RCT, In-patients, Adverse events checklist	*N* = 10, 37.58 ± 8.96 years, 80% male	*N* = 10, 37.6 ± 9.0 years, 80% male	450 ± 0	6 (10%)	6 (60%)

Table S1. *Cont.*

Study	Location, Design, Setting, and Constipation Assessment Method	Clozapine-Treated Patients, *N*, Age, % Male	Other Antipsychotics-Treated Patients, *N*, Age, % Male	Mean Clozapine Dose (mg) ± SD *	Number (%) Clozapine-Treated Patients with Constipation	Number (%) Patients with Constipation in Other Antipsychotics Group
Kluge *et al.*, 2007 [73]	Germany, RCT, In-patients, Self-reported	*N* = 15, 36.7 ± 13 years, 47% male	*N* = 15, 32.8 ± 8.3 years, 33% male	266.7 ± 77.9	3 (20%)	1 (7%)
Meltzer *et al.*, 2003 [10]	USA, Canada, UK, Italy, Croatia, France, South Africa, Argentina, Chile, Czech republic, Hungary, RCT, Out-patients, Self-reported	*N* = 479, 37.1 ± 10.3 years, 82% male	*N* = 477, 37 ± 10.3 years, 79% male	274.2 ± 155	120 (25.1%)	46 (9.6%)
Naber *et al.*, 2005 [81]	Germany, RCT, In-patients, Self-reported	*N* = 57, 35.2 ± 10.8 years, 61.4% male	*N* = 57, 32.9 ± 10.4 years, 61.4% male	209 ± 91	12 (21%)	0 (0%)
Tang *et al.*, 2011 [74]	China, RCT, In-patients, Adverse events checklist	*N* = 30, 28.6 ± 8.5 years, 61% male	*N* = 32, 28.4 ± 9 years, 60% male	No mean stated (range 200–500)	22 (73.3%)	4 (12.5%)
Tolleffson *et al.*, 2001 [77]	UK, Belgium, Italy, Finland, Spain, Germany, Denmark, France, Norway, Portugal, South Africa, Sweden, Switzerland, Ireland, RCT, Mixed in- and out-patients, Self-reported	*N* = 90, 38.6 ± 10.6 years, 0% male	*N* = 90, 38.6 ± 10.6 years, 0% male	303.6 ± 108.7	17 (18.9%)	6 (6.7%)

* In clozapine-treated patients.

Table S2. Summary of non-controlled studies included measuring the prevalence of constipation in clozapine-treated patients.

Study	Location, Design and Setting	Clozapine-Treated Patients, N, Age, % Male	Mean Clozapine Dose (mg) ± SD	Number (%) Clozapine-Treated Patients with Constipation
Aamani *et al.*, 2015 [58]	India, Prospective cohort, In-patients, Clinician diagnosis	*N* = 30, Mean age not stated, 70% males	450 ± not stated	7 (24%)
Antonacci & de Groot, 2000 [50]	USA, Retrospective cohort, In-patients, Self-reported	*N* = 33, 40.2 ± 9.7 years, 51.1% males	400 ± not stated	10 (30.3%)
Bailey, 2015 [57]	UK, Retrospective cohort, Out-patients, Laxative use	*N* = 202, 44.6 ± not stated, 69.8% males	393.8 ± not stated	71 (35%)
Centorrino *et al.*, 1994 [72]	USA, Cross-sectional, Out-patients, Adverse events checklist	*N* = 44, 36 ± 9.1 years, 75% males	294 ± 165	14 (32%)
De Leon, 2003 [17]	USA, Prospective cohort, In-patients, Adverse events checklist	*N* = 27, 44.8 ± 9.6 years, 44% males	600 ± 0	9 (33%)
Drew, 1999 [69]	Australia, Retrospective cohort, Out-patients, Adverse events checklist	*N* = 24, 34.5 ± 8.5 years, 56% males	410 ± not stated	5 (20.8%)
Dutt *et al.* , 2010 [49]	India, Prospective cohort, In-patients, Clinician diagnosis	*N* = 51, 32.1 ± 6.9 years, 80.4% males	298.97 ± 94.3	8 (15.6%)

Table S2. *Cont.*

Study	Location, Design and Setting	Clozapine-Treated Patients, N, Age, % Male	Mean Clozapine Dose (mg) ± SD	Number (%) Clozapine-Treated Patients with Constipation
Ebrahim *et al.*, 1994 [65]	USA, Prospective cohort, In-patients, Self-reported	*N* = 27, 33.8 ± 6.87 years, 100% males	558 ± 210	18 (66%)
Faasen *et al.*, 2014 [71]	South Africa, Cross-sectional, Out-patients, Self-reported	*N* = 29, 40.1 ± 9.9 years, 90% males	319.8 ± 158.3	13 (44.8%)
Golden, 2008 [62]	South Africa, RCT, Out-patients, Clinician diagnosis	*N* = 36, 39.8 ± 9.4 years, 64% males	200 ± 0	3 (8.3%)
Hayes and Gibler, 1995 [59]	USA, Retrospective cohort, In-patients, Self-reported	*N* = 53, Mean age not stated, % males not stated	Mean dose not stated	32 (60%)
Honer *et al.*, 2006 [63]	Canada, Germany, China, UK, RCT, Mixed in- and out- patients, Adverse events checklist	*N* = 33, 34.9 ± 8.5 years, 74% males	492 ± 131	7 (21%)
Hynes *et al.*, 2015 [24]	UK, Outpatients, Cross-sectional, Adverse events checklist	*N* = 110, 42 ± 10.1 years, 87% males	332 ± 134	53 (48%)
Kelly *et al.*, 2015 [64]	USA, RCT, Mixed in- and out-patients, Adverse events checklist	*N* = 23, 42.3 ± 11 years, 78% males	433.7 ± 140.1	10 (43%)

Table S2. *Cont.*

Study	Location, Design and Setting	Clozapine-Treated Patients, N, Age, % Male	Mean Clozapine Dose (mg) ± SD	Number (%) Clozapine-Treated Patients with Constipation
Kim *et al.*, 2009 [66]	South Korea, Prospective cohort, Outpatients, Self-reported	*N* = 19, 36.4 ± 7.1 years, 69.2% males	600 ± not stated	4 (21%)
Kishi *et al.*, 2013 [61]	Japan, Prospective cohort, In-patients, Clinician diagnosis	*N* = 38, 47.6 ± 2.5 years, 47.4% males	328 ± not stated	25 (65.8%)
Lieberman *et al.*, 1994 [60]	USA, Prospective cohort, In-patients, Adverse events checklist	*N* = 77, 28.2 ± 6.1 years, 67% males	458 ± 222	41(53.3%)
Lokshin, 1999 [48]	Israel, Retrospective cohort, In-patients, Self-reported	*N* = 59, 37.6 ± not stated, 67.8% males	202 ± not stated	7 (12%)
Spina *et al.*, 2000 [68]	Italy, Prospective cohort, Out-patients, Clinician diagnosis	*N* = 45, 38.1 ± 10.4 years, 78% males	309 ± 63	3 (6.7%)
Srivastava, 2002 [56]	India, Retrospective cohort, Out-patients, Adverse events checklist	*N* = 15, 30.6 ± 6.2 years, 60% males	248.21 ± not stated	1 (6.7%)
Yusufi *et al.*, 2007 [70]	UK, Cross-sectional, In-patients, Adverse events checklist	*N* = 103, 39.3 ± 8.8 years, 69% males	456 ± 209	35 (34%)
